# Isolation and characterization of a novel alphanodavirus

**DOI:** 10.1186/1743-422X-8-311

**Published:** 2011-06-19

**Authors:** Huimin Bai, Yun Wang, Xiang Li, Haitao Mao, Yan Li, Shili Han, Zhengli Shi, Xinwen Chen

**Affiliations:** 1 State Key Lab of Virology, Wuhan Institute of Virology, Chinese Academy of Sciences, Wuhan, 430071, China; 2 Graduate School of the Chinese Academy of Sciences, Beijing, 100039, China; 3 College of Life Sciences, Huazhong Normal University, Wuhan, 430079, China; 4 Department of Cell Biology, Wenzhou Medical College, Wenzhou, 325035, China; 5 Institut Pasteur of Shanghai, Chinese Academy of Sciences, Viral Hepatitis Research Unit, Shanghai, 200025, China

**Keywords:** Alphanodavirus, HzNV, Hz-AM1, Host susceptibility

## Abstract

**Background:**

*Nodaviridae *is a family of non-enveloped isometric viruses with bipartite positive-sense RNA genomes. The *Nodaviridae *family consists of two genera: alpha- and beta-nodavirus. Alphanodaviruses usually infect insect cells. Some commercially available insect cell lines have been latently infected by Alphanodaviruses.

**Results:**

A non-enveloped small virus of approximately 30 nm in diameter was discovered co-existing with a recombinant *Helicoverpa armigera *single nucleopolyhedrovirus (*Hear*NPV) in Hz-AM1 cells. Genome sequencing and phylogenetic assays indicate that this novel virus belongs to the genus of alphanodavirus in the family *Nodaviridae *and was designated HzNV. HzNV possesses a RNA genome that contains two segments. RNA1 is 3038 nt long and encodes a 110 kDa viral protein termed protein A. The 1404 nt long RNA2 encodes a 44 kDa protein, which exhibits a high homology with coat protein precursors of other alphanodaviruses. HzNV virions were located in the cytoplasm, in association with cytoplasmic membrane structures. The host susceptibility test demonstrated that HzNV was able to infect various cell lines ranging from insect cells to mammalian cells. However, only Hz-AM1 appeared to be fully permissive for HzNV, as the mature viral coat protein essential for HzNV particle formation was limited to Hz-AM1 cells.

**Conclusion:**

A novel alphanodavirus, which is 30 nm in diameter and with a limited host range, was discovered in Hz-AM1 cells.

## Background

*Nodaviridae *is a family of non-enveloped isometric viruses with a bipartite positive-sense RNA genome [1]. The *Nodaviridae *family has two genera: alphanodavirus, originally isolated from insects, and betanodavirus, one of the causal agents of mortality in cultured marine fish species worldwide [2,3]. In addition, an unclassified nodavirus, *Macrobrachium rosenbergii *nodavirus, has been isolated from *M. rosenbergii*, which is a giant freshwater prawn [4].

Nodavirus particles range from 29 to 35 nm in diameter and consist of two segments of single-stranded RNA that are co-packaged into one virion [5]. RNA1, which is approximately 3.1 kb, translates a protein termed protein A that consists of multiple functional domains: a mitochondrial targeting domain, a transmembrane domain [6,7], an RNA-dependent RNA polymerase (RdRp) domain [8], a self-interaction domain [9], and an RNA capping domain [10]. In addition, RNA1 encodes a subgenomic RNA3 that translates protein B2, an RNA silencing inhibitor [11-14]. RNA2 encodes protein α, a viral capsid protein precursor, which is auto-cleaved into two mature proteins, a 38 kDa β protein and a 5 kDa γ protein, at a conserved Asn/Ala site during virus assembly [15].

Alphanodavirus TNCL (Tn5 cell line virus) have been shown previously to latently infect a commercially available Hi5 cell line, with the viral coat protein induced by the presence of recombinant baculoviruses [16]. In this report, an unidentified non-enveloped small virus of about 30 nm in diameter was discovered in Hz-AM1 cells co-existing with the recombinant *Helicoverpa armigera *single nucleopolyhedrovirus (*Hear*NPV) [17]. Genome sequencing and phylogenetic assays indicate that this unidentified virus belongs to the alphanodavirus genus and has been designated HzNV (Hz-AM1 derived nodavirus).

## Methods

### Cell culture and virus infection

Insect cell lines Hz-AM1 [18] and Sf9 [19] were maintained in Grace's medium (Invitrogen) supplemented with 10% fetal bovine serum (FBS) (Invitrogen) at 27°C. Baby hamster kidney (BHK) cells were cultured in DMEM (Invitrogen) with 10% FBS at 37°C. Cotton Bollworm (*H. armigera*) larvae were grown and infected with recombinant *Hear*NPV as previously described [20].

Fresh cells grown in monolayer were infected with either virus stock (including *H. armigera *hemolymph or purified virus stock) or mock virus. The viral supernatant was removed after a 2 h incubation to allow virus attachment and entry into host cells. The infected cells were then rinsed twice with serum-free medium and replenished with complete medium to support cell growth and virus replication.

### Virus purification

The hemolymph of recombinant *Hear*NPV-infected *H. armigera *larvae were used to infect fresh Hz-AM1 cells (1 × 10^8^). At 7 dpi, the viral supernatant was harvested and centrifuged at 10,000 × g for 20 min to remove cell debris. The pre-cleared supernatant was centrifuged at 120,000 × g for 2.5 h at 4°C with a 20% (w/w) sucrose cushion, and the subsequent precipitates were resuspended in 200 μl 0.1 M TE buffer (10 mM Tris.HCl, 1 mM EDTA, pH = 7.6). The enriched virus stock was further purified using either a continuous sucrose gradient or CsCl centrifugation. For sucrose-based purification, virus stock was laid on top of a 10% to 50% (w/w) continuous sucrose gradient and centrifuged at 180,000 × g for 2 h at 4°C. The banded virus particles were collected and resuspended in 0.1 M TE buffer. For CsCl gradient centrifugation, 2.1 g CsCl was dissolved in 4.5 ml virus stock and centrifuged at 32,000 rpm for 24 h at 10°C with an SW55 rotor. The banded virus was collected and enriched by 32,000 rpm for 3 h at 4°C with an SW40 rotor. The resultant precipitates were dissolved in 0.1 M TE buffer.

### Transmission electron microscopy (TEM)

Fresh Hz-AM1 cells (1 × 10^6^) were infected with either hemolymph from *H. armigera *larvae bearing recombinant *Hear*NPV or purified virus stock, and harvested at 72 hpi. The infected cells were fixed in 2.5% glutaraldehyde for 3 h at 4°C, and further treated with 1% osmic acid for 2 h. The samples were dehydrated and embedded in Epon Ultra-thin sections, were taken and stained with uranyl acetate and lead citrate.

For negative staining, purified virions were attached onto a carbon-coated grid for 5 min at room temperature. The grid was rinsed with distilled water and stained with 1% phosphotungstic acid for 3 min before air drying on filter paper.

All the specimens were viewed using a Tecnai G2 transmission electron microscopy at 75 kV.

### Western blot assay

Cell lysates containing 20 μg of total protein from virus infected cells were separated on a 12% sodium dodecyl sulfate polyacrylamide gel (SDS-PAGE) by electrophoresis and subjected to western blot assay. The proteins were transferred to a membrane that was blocked in 5% bovine serum albumin (BSA, Merck) for 1 h at room temperature and incubated with the anti-TNCL coat protein IgG (Provided by Dr. Tian-Cheng Li, National Institute of Infectious Diseases, Japan) or anti-ERK (CST) overnight at 4°C, followed by extensively washing with TBST (50 mM Tris.HCl, pH = 7.4, 150 mM NaCl, 0.1% Tween 20). The membrane was then incubated with the HRP-conjugated goat anti-rabbit secondary antibody (Abcam) for 1 h at room temperature before being developed with West Pico ECL reagent (Pierce).

### Viral gene amplification and full-length genome cloning

The virus genome was extracted from the purified virus stock using the TIANamp virus DNA/RNA Kit (Tiangen Biotech) according to the manufacturer's protocol. The viral genome was reverse transcribed using 200 units of M-MLV (Promega) in a 20 μl reaction volume with the anchored random primer (5'-GCCGGAGCTCTGCAGAATTCNNNNNN-3') at 37°C for 1 h to generate a viral cDNA pool. Double-stranded DNA was synthesized at 37°C for 30 min in the 20 μl reverse transcription reaction by adding 3 μl Klenow fragment buffer, 10 pmol of universal primer-dN6 and 8 units of Klenow fragment (Promega). Random PCR was conducted in a 50 μl volume containing 10 μl PCR buffer, 1 mM MgSO4, 0.2 mM of each dNTP, 20 pmol anchor primer (5'-GCCGGAGCTCTGCAGAATTC-3'), 1 unit of KOD-plus DNA polymerase (Toyobo), and 2 μl double-stranded DNA. The reaction was performed for 40 cycles of 94°C/30 s, 54°C/30 s, 68°C/2 min followed by incubation for 10 min at 68°C. The final PCR products were extracted, cloned into pGEM-Teasy vectors (Promega), and submitted for sequencing.

The full-length viral genome was cloned by RACE, using the sequenced viral gene fragments acquired from the previous cloning step. The RACE experiment was performed using the 3' and 5' RACE System (Invitrogen) according to the manufacturer's instructions.

### Host susceptibility assay

Hz-AM1, Sf9, and BHK were infected with equal aliquots of either virus stock or mock-virus. The infected cells were harvested at 6 dpi. Total RNAs were extracted by using TRIzol reagent (Invitrogen) according to the manufacturer's protocol, and 2 μg of total RNA from each sample were used as the template for reverse transcription with M-MLV (Promega) and random primers. The subsequent cDNAs of each sample were amplified by PCR with primer BH4-F (5'-GGAAAGTACCCATCCAGATGTCCAC-3') and BH4-R (5'-CGTGGCGTTGGGAGTGGGG-3'), which covered a 413-nt fragment of the sequenced viral genome. The PCR products were separated by gel electrophoresis on a 0.7% agarose gel. For the western blot assay, samples from the infected cells were treated as described previously.

### Bioinformatical analysis and sequence accession numbers

The viral nucleotide and predicted amino acid sequences obtained through ORF finder (http://www.ncbi.nlm.nih.gov/projects/gorf/) were submitted to BLAST analysis to retrieve homologous sequences [21]. Based on the predicted amino acid sequence of the virus coat protein precursor, MEGA 4.0 software was employed to generate a phylogenetic tree using the neighbor-joining method with 1000 bootstrap replications [22-24].

The coding sequences of HzNV coat protein precursor and protein A have been deposited in GenBank under the accession numbers GU976286 and GU976287.

## Results

### Viral morphology and phenotype

**Figure 1:**
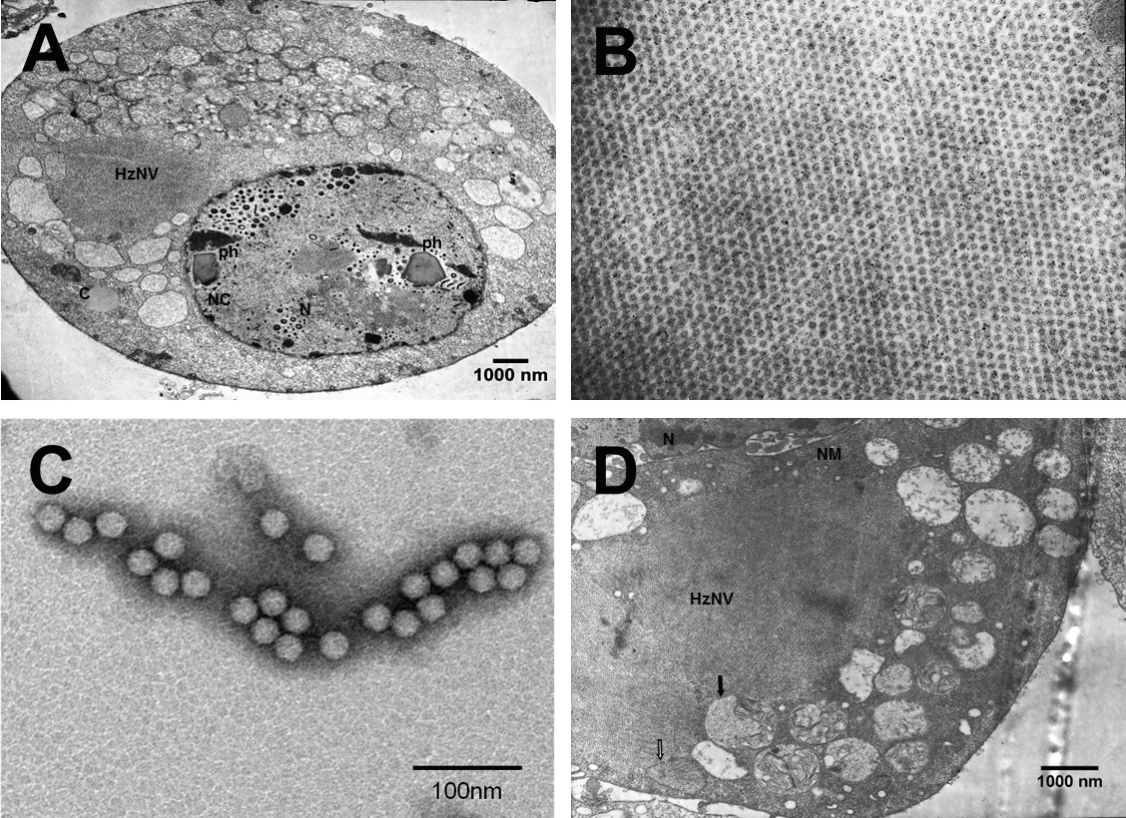
**Electron microscopic study of HzNV**. (A) Co-existence of HzNV and *Hear*NPV in Hz-AM1 cells. Fresh Hz-AM1 cells grown in monolayer were infected with *H. armigera *hemolymph bearing recombinant *Hear*NPV. Cells were processed for TEM at 2 dpi. N: nuclear; C: cytoplasm; NC: nuclear capsid; ph: polyhedra. (B) Crystal-arrayed HzNV in the cytoplasm of Hz-AM1. Hz-AM1 cells were infected with purified HzNV and processed for TEM at 3 dpi. (C) Negative staining of purified HzNV virions. (D) Membrane structures were joint with crystal array of HzNV in the cytoplasm of HzNV infected Hz-AM1 cell. Hz-AM1 cells were infected with HzNV. At 2 dpi, cells were processed for TEM. The vacuole indicated by a solid arrow displayed some residual membrane structures, while the vacuole indicated by the open arrow was filled with HzNV particles with no structure. N, nuclear, NM, nuclear membrane.

When the hemolymph of *H. armigera *larvae, bearing recombinant *Hear*NPV, were used to infect fresh Hz-AM1 cells, an array of non-enveloped, small sized and spherical viral particles were seen in addition to the expected NPV pathology by electron microscopy (TEM) (Figure 1A). These non-baculovirus virions were predominantly located in the cytoplasm and were arrayed in a crystal lattice pattern (Figure 1B). TEM assay of the negatively stained purified viral particles revealed that the non-enveloped virions exhibited a mean diameter of 30 nm (Figure 1C). Detailed observation of the Hz-AM1 cells at the early infection stage (2 days post infection (dpi)) showed the virions were located within membrane-bound spherules in the cytoplasm (Figure 1D).

These morphological features suggest that the unidentified virus possibly belongs to the group of positive-strand RNA viruses that are usually associated with host cytoplasmic membranes (reviewed in [25]). A nuclease digestion assay demonstrated that the purified viral genome was hydrolyzed by RNase A, but not by DNase I (data not shown), suggesting that the unidentified virus possesses an RNA genome.

Through CsCl gradient centrifugation, the unidentified virus was enriched at a layer of approximately 1.346 g/cm^3 ^with respect to density. This finding is similar to the CsCl buoyant density of the TNCL virus (approximately 1.350 g/cm^3^) [16].

### Identification of HzNV by western blot and RT-PCR analyses

**Figure 2:**
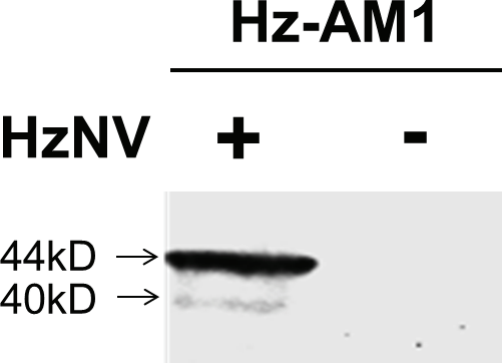
**Western blot of HzNV or mock infected Hz-AM1 cells**. HzNV and mock viruses were used to infect Hz-AM1 cells. At 3 dpi a western blot assay was performed on the cells using an anti-TNCL antibody at a dilution factor of 1:10000.

The morphological and physical characteristics combined with the knowledge that alphanodavirus can latently infect insect cells [16] prompted us to perform a western blot assay to determine whether this unidentified virus could serologically cross-react with anti-TNCL, which is an antibody that recognizes the TNCL virus coat protein [16]. A major band of about 44 kDa, and a minor band of approximately 40 kDa were detected by western-blot in the cell lysate of Hz-AM1 cells infected with the purified virus (Figure 2). In contrast, the mock-infected Hz-AM1 cells exhibited no serological cross-reaction with anti-TNCL (Figure 2). This cross-reactivity indicates that the virus encodes a viral protein that shares sequence homology with the coat protein of alphanodavirus.

To further characterize this virus at the nucleotide level, reverse transcription-polymerase chain reaction (RT-PCR) was used to identify the viral gene sequence information. Random PCR from the viral cDNA pool produced a smear of bands that were subsequently extracted and cloned into pGEM-Teasy vectors for sequencing. Sequence analysis revealed two homologous fragments of nodavirus according to a BlastX search [21]; a 447-bp fragment encoding a translated product with 35% amino acid identity with the Pariacoto virus protein A (Genbank: NP_620109.1) (data not shown), and a 454-bp fragment encoding a viral protein with 58% identity with protein α of flock house nodavirus (FHV, Genbank: NP_689442.1) (data not shown). Therefore, the gene sequencing results and the viral protein antigenic properties indicates that the unidentified virus found in the Hz-AM1 cell is a new member of Nodaviridae. We have designated the virus HzNV.

### Genomic organization and bioinformatical analysis of HzNV

Because full-length genome information is essential for the detailed classification and phylogenetic analysis of HzNV, rapid amplification of cDNA ends (RACE) was performed with sequences identified by RT-PCR to clone the two full-length HzNV RNA fragments. RNA1 of HzNV is 3038 nt long, contains a 71 nt 5' Untranslated Region (UTR) and a 15 nt 3'UTR, and could be computationally translated into a 983-aa protein that exhibits homology (41%-55%) with protein A from a variety of nodaviruses (data not shown).

The full-length HzNV RNA2 is 1404 nt and putatively encodes a 408-aa capsid protein α. This protein shows homology to various degrees with other members of the Nodaviridae family, including Black beetle virus (BbV), Boolarra virus (BoV), Nodamura virus (NoV), and Pariacoto virus (PaV) (Table 1). The predicted molecular mass of the HzNV capsid protein is 44 kDa, which is equivalent to the molecular mass of the major band seen by western blot assay (Figure 2). Analysis of the predicted amino acid sequence encoded by HzNV RNA 2 revealed conserved protein α cleavage sites located at ^363^Asn and ^364^Ala. If cleavage occurs at these sites, the resulting protein, protein β, would have a molecular mass of 40 kDa, which corresponds to the minor band seen by the western blot assay (Figure 2).

**Table 1:** Protein A and coat protein precursor (protein α) homology among Alphanodavirus

ProA Pro α	HzNV	FHV	NoV	BBV	PaV	BoV	TNCL
HzNV		59%	48%	62%	37%	53%	61%
FHV	43%		51%	87%	38%	52%	89%
NoV	43%	58%		51%	36%	44%	51%
BBV	42%	90%	57%		37%	53%	83%
PaV	55%	38%	41%	38%		37%	35%
BoV	41%	90%	58%	90%	42%		50%
TNCL	41%	97%	57%	97%	39%	90%	

**Figure 3:**
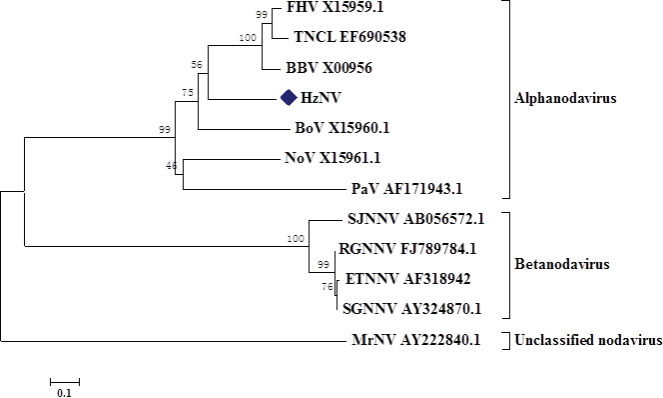
**Phylogenetic tree of HzNV based on the sequence of the coat protein precursor**. The predicted amino acids of the coat protein precursor were used to generate a phylogenetic tree by MEGA 4 software with the neighbor-joining method. The bootstrap equals 1,000 replications. The relevant GenBank accession numbers are indicated in the graph. FHV: flock house virus; BBV: Black beetle virus; BoV: Boolarra virus; NoV: Nodamura virus; PaV: Pariacoto virus; TNCL: Tn-5 drived nodavirus; SJNNV: *Striped jack *nervous necrosis virus; RGNNV: Red-spotted Grouper Nervous Necrosis Virus; ETNNV: *Epinephelus tauvina *nervous necrosis virus; SGNNV: sevenband grouper nervous necrosis virus; MrNV: *Macrobrachium rosenbergii *nodavirus.

BBV: Black beetle virus; BoV: Boolarra virus; NoV: Nodamura virus;

PaV: Pariacoto virus;

To determine the relationship between HzNV and other nodaviruses, with respect to evolutionary distance, a phylogenetic tree was generated by comparing protein α of HzNV with other nodaviruses. The results indicate that HzNV is more closely related to alphanodavirus than to betanodavirus (Figure 3). Within the alphanodavirus clade, HzNV is the closest to the black beetle virus in the alphanodavirus genera.

### Maturation of HzNV coat protein and secretion dynamics of virions

**Figure 4:**
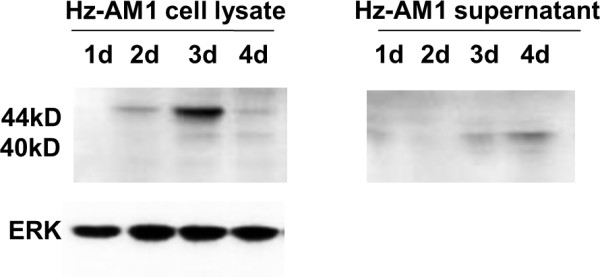
**Virus coat protein maturation and virion secretion**. Hz-AM1 cells and supernatants infected with HzNV were collected each day post infection. The maturation of coat protein (protein precursor cleavage) was tracked by an anti-TNCL antibody.

To characterize the time course of HzNV coat protein maturation and virus propagation dynamics, a western blot assay using anti-TNCL antibody was performed at various time points after infection (Figure 4). The western blot results demonstrate that the HzNV capsid protein precursor (44 kDa) can be detected as early as 2 dpi and was further cleaved into the mature form (40 kDa) from 3 dpi. This finding is consistent with the appearance of secreted mature capsid protein in the supernatant (Figure 4). This also suggests that *de novo *synthesized HzNV particles can be secreted into the medium and that Hz-AM1 cells are fully permissive for HzNV.

### Examination of HzNV latent infection and range of hosts

**Figure 5:**
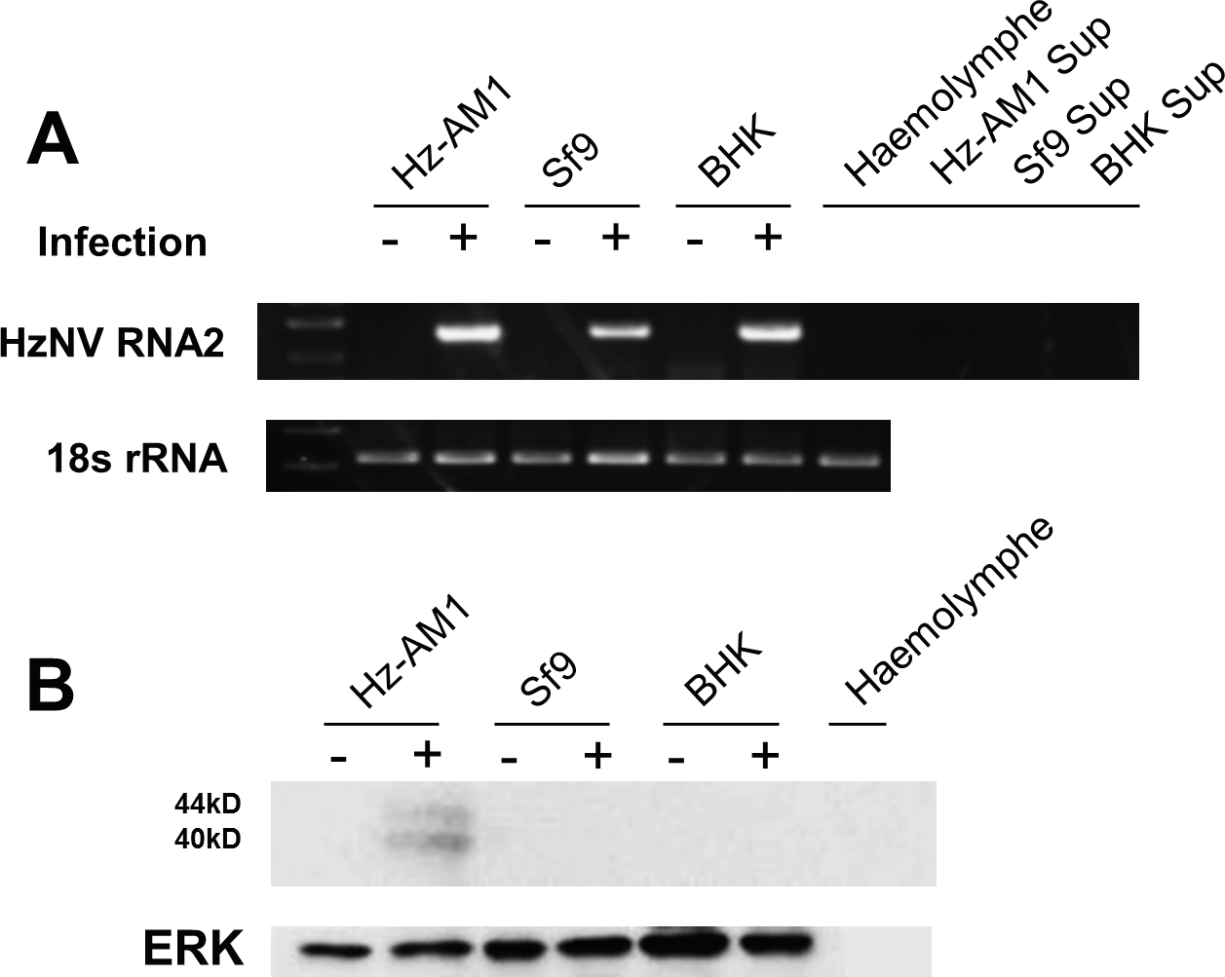
**Examination of HzNV host cell susceptibility**. Hz-AM1, Sf9, and BHK21 cells were infected with HzNV at equal amounts. At 6 dpi, the cell lysates, cell supernatant (sup), and hemolymph of healthy insect larvae (baculovirus-free) were used for RT-PCR detection of HzNV-RNA2 fragment, respectively. The primer set BH4F/R was used to amplify a specific HzNV fragment, and the *18s *rRNA gene was used as an internal control. (B) Cells and haemolymph indicated in (A) were used for a western blot assay using an anti-TNCL antibody. ERK was used as the internal control.

Nodaviruses reportedly cause unapparent, latent infection in their hosts and have a relatively wide host range [16,26-28]. RT-PCR and western blot analyses were performed to analyze HzNV latent infection and permissiveness in various cell lines. Using a HzNV-specific primer set, a 413-bp fragment was found in all the HzNV-infected cell lines including Hz-AM1, Sf9, and BHK, but it was absent from all un-infected cells. This finding indicates that HzNV is infectious to all the cell lines tested, and no latent infection existed (Figure 5A). By western blot assay, viral capsid protein precursor (44 kDa) and mature form (40 kDa) were only detected in virus-infected Hz-AM1 cells (Figure 5B), indicating that HzNV can only generate viral structural proteins in Hz-AM1; no coat protein or *de novo *viral particles were synthesized in the other cell lines tested even though HzNV was infectious to these cell lines.

To investigate whether HzNV pre-existed in a natural host, the hemolymph of healthy *H. armigera *(baculovirus-free) was used for RT-PCR with HzNV-specific primers and probed with anti-TNCL. Neither experiment displayed the presence of HzNV (Figure 5A and 5B), which indicates that the origin of HzNV may be attributed to an accidental contamination of Hz-AM1 cells with HzNV when recombinant *Hear*NPV bacmid was transfected into Hz-AM1 cells. The HzNV was further propagated when the contaminated *Hear*NPV was injected into *H. armigera *larvae.

## Discussion

In this report, a non-enveloped isometric virus approximately 30 nm in diameter was discovered co-existing with *Hear*NPV in Hz-AM1 cells. The virions were associated with cytoplasmic membrane structures in a manner resembling the subcellular distribution pattern of positive-stranded RNA viruses (reviewed in [25]). By virus genome sequencing and bioinformatical analysis, this novel virus was identified as a new member of alphanodavirus, and it was designated HzNV.

Genome replication of positive-stranded RNA virus depends on intracellular membrane structures, e.g., equine arteritis virus (EAV) induces endoplasmic reticulum-derived double-membrane vesicles [29]; alphavirus RNA replication factor is located on the cytoplasmic surface of endosomes and lysosomes [30]; and peripheral vesicles are present in clusters near the surface of the chloroplast surface in turnip yellow mosaic virus (TYMV) infected Chinese cabbage leaves [31]. For nodavirus, FHV RNA replication factor and *de novo *synthesized viral RNA are distributed predominantly within infection-induced membrane spherules inside the outer mitochondrial membrane with a necked channel connected to the cytoplasm [32]. In addition to FHV, Atlantic halibut nodavirus (AHNV), and carnation Italian ringspot virus (CIRV) all possess a mitochondrial localization signal (MLS) on protein A that relocates the viral RNA replication factor to mitochondria [33-35].

In our study, HzNV was also associated with amorphous cytoplasmic membrane structures in the early stage of infection. HzNV protein A was computationally predicted to localize within mitochondria, suggesting that the virus-induced membrane structures are possibly derived from mitochondria. The mature virions formed crystal arrays in the cytoplasm at the late stage of infection. This finding suggests that the HzNV maturation process resembles that of Nodamura virus (NoV), which is a member of the alphanodavirus genus, whose genome replication factor is associated with the mitochondrial membrane and whose virions also form crystal arrays in the cytoplasm during the late phase of infection [36,37].

Nodaviruses often cause unapparent, latent infections in their hosts and have a relatively wide host range [16,26-28]. A latent infection of TNCL virus in commercially available Hi5 cells was previously reported [16,26], in which the TNCL viral genome was detected in fresh cells, and baculovirus co-infection triggered viral coat protein expression [16]. In the present study, HzNV is infectious to Hz-AM1, Sf9, and BHK cells. However, only Hz-AM1 appeared to be fully permissive to HzNV; HzNV-infected Hz-AM1 cells harbored viral coat protein, which is the structural protein essential for production of *de novo *HzNV particles.

Insect cell lines are widely used in baculovirus expression vector systems (BEVS) for production of recombinant protein and virus-like particles frequently used in vaccine development. Therefore, the latent infection or accidental contamination of alphanodaviruses such as the TNCL virus or HzNV should raise the safety concerns about the application of insect cell lines for medical purposes.

## Conclusions

A novel alphanodavirus, HzNV, was isolated and characterized. The host susceptibility test indicates that Hz-AM1 cells are fully permissive for HzNV, although HzNV is infectious to a variety of cell types ranging from insect cells to mammalian cells.

## Competing interests

The authors declare that they have no competing interests.

## Authors' contributions

HB generated much of the data and co-wrote the manuscript. XC and YW designed the experiments and co-wrote the paper. XL and HM were involved in the preliminary work discovering HzNV in the Hz-AM1 cells. YL and ZS cloned the partial sequence of HzNV. SH assisted with infection and performed some of the western blots. All authors read and approved the final manuscript.
